# Association between the lean nonalcoholic fatty liver disease and risk of incident type 2 diabetes in a healthy population of Northwest China: a retrospective cohort study with a 2-year follow-up period

**DOI:** 10.3389/fendo.2023.1173757

**Published:** 2023-06-26

**Authors:** Nong Li, Weiting Xang, Shengli Wu, Danting Li, Min Chang, ChengYao Xie, Mei Yu Zhang, Huiwen Tan

**Affiliations:** ^1^ Department of Endocrinology and Metabolism, the Hospital of Integrated Traditional Chinese Medicine and Western Medicine of Karamay, Xinjiang, China; ^2^ Department of Health Management Center, West China Hospital, Sichuan University, Chengdu, Sichuan, China; ^3^ Department of Health Management Center, the Hospital of Integrated Traditional Chinese Medicine and Western Medicine of Karamay, Xinjiang, China; ^4^ Department of Endocrinology Metabolism, West China Hospital of Sichuan University, Chengdu, China

**Keywords:** nonalcoholic fatty liver disease, risk factor, type 2 diabetes, visceral fat obesity, cohort study

## Abstract

**Aims:**

We aimed to explore the metabolic features of lean nonalcoholic fatty liver disease (Lean-NAFLD) and its association with the risk of incident type 2 diabetes in young and middle-aged people.

**Methods:**

We conducted a retrospective cohort study of 3001 participants who were enrolled in a health check-up program from January 2018 to December 2020 in the Health Management Center of Karamay People’s Hospital. The age, sex, height, weight, body mass index (BMI), blood pressure, waist circumference (WC), fasting plasma glucose (FPG), lipid profiles, serum uric acid and alanine aminotransferase (ALT) of the subjects were collected. The cutoff point of BMI for lean nonalcoholic fatty liver disease is <25 kg/m^2^. A COX proportional hazard regression model was used to analyze the risk ratio of lean nonalcoholic fatty liver disease to type 2 diabetes mellitus.

**Results:**

Lean NAFLD participants had many metabolic abnormalities, such as overweight and obesity with nonalcoholic fatty liver disease. Compared with lean participants without nonalcoholic fatty liver disease, the fully adjusted hazard ratio (HR) for lean participants with nonalcoholic fatty liver disease was 3.83 (95% CI 2.02-7.24, p<0.01). In the normal waist circumference group (man<90cm, woman<80 cm), compared with lean participants without NAFLD, the adjusted hazard ratios (HRs) of incident type 2 diabetes for lean participants with NAFLD and overweight or obese participants with NAFLD were 1.93 (95% CI 0.70-5.35, p>0.05) and 4.20 (95% CI 1.44-12.22, p<0.05), respectively. For excess waist circumference (man≥90 cm, woman ≥80 cm) compared with lean participants without NAFLD, the adjusted hazard ratios (HRs) of incident type 2 diabetes for lean participants with NAFLD and overweight or obese participants with NAFLD were 3.88 (95% CI 1.56-9.66, p<0.05) and 3.30 (95% CI 1.52-7.14, p<0.05), respectively.

**Conclusion:**

Abdominal obesity is the strongest risk factor for type 2 diabetes in lean nonalcoholic fatty liver disease.

## 1 Introduction

At present, NAFLD has become one of the most common liver diseases affecting the health condition of adults and children in the world (1, 2) and has brought a huge burden to the global health care system. Approximately 25% of the global population is affected by NAFLD, and Middle Eastern countries and South America have the highest incidence of NAFLD in the world (3, 4). NAFLD is characterized by the accumulation of more than 5% fat in hepatocytes (5), which includes hepatic steatosis, steatohepatitis and liver fibrosis, and further development of the lesions can lead to cirrhosis and hepatocellular carcinoma (6) NAFLD is a multisystem disease that increases the risk of type 2 diabetes mellitus (T2DM), cardiovascular disease (CVD), some types of extrahepatic malignancies, and chronic kidney disease (CKD), and the magnitude of this risk parallels the severity of NAFLD (especially the stage of liver fibrosis) (7, 8).

NAFLD is closely related to type 2 diabetes mellitus. NAFLD and T2DM often coexist and act synergistically, increasing the risk of hepatic and extrahepatic adverse clinical outcomes (1). T2DM is also one of the strongest risk factors for faster progression of NAFLD to nonalcoholic steatohepatitis, advanced fibrosis, or cirrhosis (T2DM plays an important role in disease progression to NASH, liver fibrosis, and cirrhosis). The global prevalence of NAFLD in patients with T2DM was 55.5% (95% CI: 47.3-63.7) (9) more than half of T2DM patients have been diagnosed with NAFLD, and there is a strong correlation between them. Obesity, physical inactivity and metabolic syndrome are common risk factors (1, 10–12).

Nonalcoholic fatty liver disease (NAFLD) can be classified into lean or nonoverweight obese (BMI < 25 kg/m^2^) and overweight obese (BMI≥25 kg/m^2^) according to BMI (2, 13). A systematic review and meta-analysis reported in 2020. The prevalence rates of lean NAFLD and nonobese NAFLD in the general population are 5.1% and 12.1%, respectively. In the NAFLD population, lean NAFLD and nonobese NAFLD accounted for 19.2% and 40.8%, respectively (14). Studies have shown that not only overweight and obese NAFLD may have liver and extrahepatic complications. “Lean” or “nonobese” patients with nonalcoholic fatty liver disease (NAFLD) also have hepatic and extrahepatic complications, suggesting that metabolic phenotype is more important than the clinical classification of body mass index in the prognostic assessment of NAFLD (15). However, to date, the characteristics of the lean NAFLD population are still unclear, and there are still few studies on the prevalence and outcome of lean nonalcoholic fatty liver disease based on race (16).

Studies have shown that there is a bidirectional interaction between NAFLD and type 2 diabetes (12, 17). However, the direct relationship between NAFLD and the incidence of type 2 diabetes is still less studied, and the causal relationship between the two is still unclear, especially the association between “lean” or “nonobese” nonalcoholic fatty liver disease (NAFLD) and the incidence of type 2 diabetes. Further studies are needed (2) Compared with overweight and obese NAFLD, the incidence of type 2 diabetes in people with “lean” or “nonobese” nonalcoholic fatty liver disease is also less studied worldwide, especially in China. This retrospective cohort study was conducted to investigate the association between lean nonalcoholic fatty liver disease (NAFLD) and the risk of type 2 diabetes mellitus (T2DM) in healthy people undergoing physical examination in Karamay, Northwest China.

## 2 Materials and methods

### 2.1 Subjects (study design and study participants)

In Karamay, Northwest China, the Xinjiang Oilfield Company organizes a medical health checkup program for employees and citizens every year. The medical examinations were carried out at the Medical Examination Centre of Karamay People’s Hospital. This study is a retrospective cohort study. Adults who underwent annual physical examination in the Health Management Center of Karamay People’s Hospital of Xinjiang from January 1, 2018, to December 31, 2020, were selected as the study population. The inclusion criteria were as follows: (1) Participants participated in the annual physical examination (baseline examination) at the Health Examination Center of Karamay People’s Hospital from January 2018 to December 2018. (2) Age ≥20 years, no history of diabetes. (3) Participation in annual employee health check-ups in 2019 and 2020. The exclusion criteria for subjects of study were as follows:(1) Queer alcohol intake (male>30 g/day, female>20 g/day); (2) Combined with viral hepatitis, drug-induced liver disease, hepatolenticular degeneration, autoimmune liver disease and other specific diseases that can lead to fatty liver; (3) Baseline examination, fasting blood glucose ≥ 6.1 mmol/L; (4) Loss of fasting blood glucose during baseline examination or physical examination follow-up (5); Loss of abdominal ultrasound and other parameter data during physical examination follow-up.

**Figure 1:**
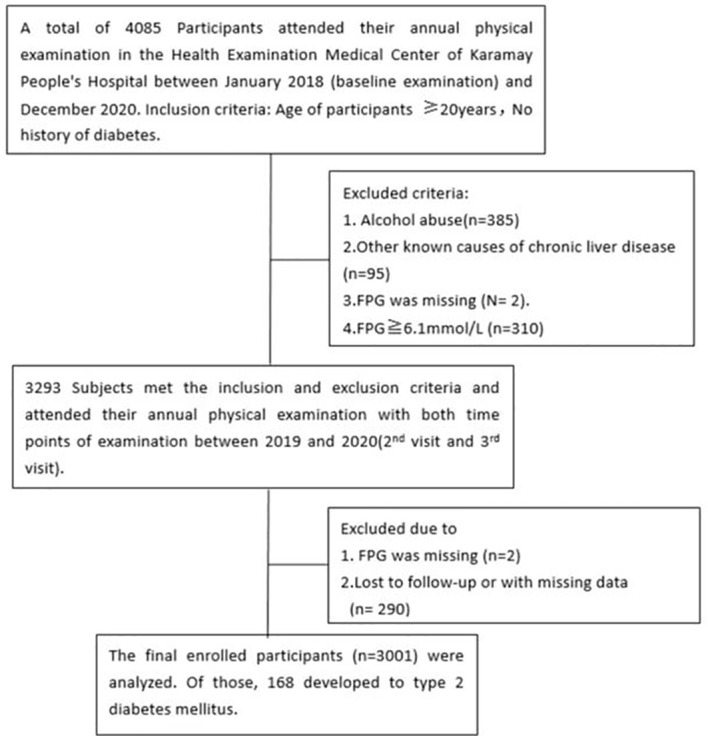
Flowchart of the present observational cohort study.

A total of 4085 people participated in the baseline examination at the Health Examination Center of Karamay People’s Hospital in 2018 and the annual employee health examination follow-up in 2019 and 2020. Based on the inclusion and exclusion criteria, a total of 3001 participants were included in the cohort analysis (see **Figure 1**). This research project follows the Helsinki Declaration and China’s clinical research management norms and regulations. The research plan was approved by the Medical Ethics Committee of Karamay People’s Hospital. Informed consent was obtained from all participants.

### 2.2 Baseline data collection and measurement

Sex, age, ethnicity, height, weight, BMI, blood pressure, waist circumference and past medical history were collected by the investigators. The subjects’ height and weight were measured in an overnight-fasted state, shoes were removed, light clothes were worn, and the readings were accurate to 0.5 kg and 0.5 cm, respectively. Body mass index (BMI) was calculated as body weight (kg) divided by squared height (m^2^) (kg/m^2^). Waist circumference (WC) was taken as the circumference of the midpoint line between the lowest point of the rib and the upper edge of the iliac crest under normal breathing conditions. Fasting blood glucose (FPG), alanine aminotransferase (ALT), triglyceride (TG), total cholesterol (TC), high-density lipoprotein cholesterol (HDL-_C_), low-density lipoprotein cholesterol (LDL-_C_) and blood uric acid (BUA) were collected. The triglycerides and glucose index (TyG) were calculated as ln (fasting TG (mg/dL) ×FPG (mg/dL)/2) (18).

### 2.3 Ultrasound examination and diagnosis of NAFLD

Abdominal ultrasound examination was performed on subjects using a color Doppler ultrasound diagnostic instrument E9 (GE Company, USA) with a transduce of 3.5 MHz. All subjects were diagnosed with fatty liver according to the results of ultrasound examination. Inspectors of the clinical information of the subjects, according to the subjects of liver tissue echoes, the differences between the liver and right kidney and blood vessels of the structure of the visibility diagnosis, ultrasonic tip liver frontcourt echogenicity (“bright liver “), the far field echo attenuation, and the display are not clear, such as structural characteristics of the intrahepatic duct in the exclusion of alcohol, virus, autoimmune, drugs and other causes of fatty liver. The by experienced sonographers (19, 20).

### 2.4 Endpoint and diagnosis of type 2 diabetes

The outcome event (study endpoint) was the onset of type 2 diabetes mellitus during the annual health check-up from 2019 to 2020. Survival was defined as the time from January 2019 to the date of diagnosis of type 2 diabetes at physical examination and was censored at the last follow-up physical examination in 2020 or at the last follow-up physical examination in 2020 without diabetes. Diabetes was diagnosed according to the 1999 World Health Organization (WHO) criteria: diabetes mellitus, fasting blood glucose ≥7.0 mmol/L, oral glucose tolerance test (OGTT) 2-hour postprandial blood glucose (2hPG) ≥11.1 mmol/L, or self-reported use of hypoglycemic drugs. Prediabetes: 6.1 mmol/L ≤ fasting glucose ≤7.0 mmol/L is impaired fasting glucose (IFG), and 7.8 mmol/L ≤2 hPPG ≤ 11.1 mmol/L is impaired glucose tolerance (IGT). Normal blood glucose: fasting blood glucose ≤6.1 mmol/L and OGTT 2hPPG ≤ 7.8 mmol/L (21).

### 2.5 the category used to define BMI and WC groups of NAFLD

The 3,001 participants were divided into four groups based on whether they were overweight/obese and NAFLD. The four groups were non-overweight/obese group without NAFLD (n = 1398), non-overweight/obese group with NAFLD (n = 160), overweight or obese group without NAFLD (n = 758), overweight or obese group with NAFLD (n = 685). BMI ≥ 25kg/m2 was defined as overweight/obese. WC≧ 90 cm in men,and WC≧≥ 80 cm in women was defined as abdominal obesity (22).

### 2.6 Statistical analysis

Excel 2007 was used to establish the database and manage the data, double input the data and correct the errors. SPSS 22.0 statistical package (IBM, Armonk, New York) was used for data processing for all statistical analyses. A normality test was performed on continuous variables of measurement data. Measurement data with a normal distribution are expressed as the mean ± standard deviation ( $\bar{\text{x}}$ ±s), and continuous data with a skewed distribution are expressed as the median and interquartile range (IQR). The Kruskal−Wallis H test or Mann−Whitney U test was used for comparisons among groups. Categorical variables are expressed as percentages. The chi-square test was used to compare categorical variables. The 3001 participants were divided into four groups based on the presence or absence of overweight and NAFLD. Taking lean subjects without NAFLD as the reference group (compared with lean subjects without NAFLD), a COX proportional hazards regression model was used to analyze overall overweight (or obesity) without NAFLD, lean with NAFLD and overweight (or obesity) with NAFLD, and abdominal obesity (WC ≥ 90 cm in men and ≥ 80 cm in women) and nonabdominal obesity subgroups were associated with the risk of type 2 diabetes, and their hazard ratios and 95% confidence intervals were calculated. Hazard ratios (HRs) with 95% confidence intervals (CIs) for the incidence of diabetes were calculated for each study phenotype using Cox proportional-hazard regression models, with lean subjects without NAFLD as the reference group. The Kaplan−Meier method was used for survival analysis to draw the risk function curves of the above four categories of type 2 diabetes, and the log-rank test was performed to compare whether there was a difference in the risk of type 2 diabetes among the four groups. The Stata 17.0 was used to plot the figure of cumulative hazard estimates. The difference was statistically significant with a P value of <0.05 (two-tailed).

## 3 Results

### 3.1 Baseline clinical characteristics of subjects

A total of 3001 subjects were enrolled in the study. The average age of these people was 43 (34-49) years. The BMI was 24.84(22.55-27)kg/m^2^, and there were 2255 men (75.1%) and 746 women. Of these, 845 had nonalcoholic fatty liver disease, while 2156 subjects had no NAFLD. A total of 81.1% of those with nonalcoholic fatty liver disease were overweight or obese, and 35.2% of those without NAFLD were overweight or obese. The number of subjects with lean nonalcoholic liver was 160, and the average BMI of these subjects was 23.86(23.05-24.48)kg/m^2^. The number of subjects with overweight or obesity with nonalcoholic liver was 685, and the average BMI of these subjects was 28.65 (26.96-30.88)kg/m^2^. In both the overweight (or obesity) with NAFLD group and the lean with NAFLD group, the baseline levels of fasting blood glucose, triglycerides, total cholesterol, low-density lipoprotein cholesterol, alanine aminotransferase, blood uric acid and TyG index were higher than those of any group without NAFLD, while the high-density lipoprotein cholesterol was lower than that of any group in the without NAFLD group (**Table 1**).

**Table 1:** Comparison of baseline characteristics of four groups from the subjects with NAFLD and the subjects without NAFLD.

Parameters	Total	Lean without NAFLD	Over- weight/Obesity without NAFLD	Lean with NAFLD	Over- weight/Obesity with NAFLD	H/X^2^	P value
Number of subjects	3001	**1398**	**758**	160	685		
Age(year)	43(34-49)	42(33-48)	45(36-51)	45(36-50)	41(34-49)	45.477	<0.001
Male, N (%)	2255(75.1%)	875(62.6%)	626(82.6)	136(85.0%)	618(90.2%)	232.1	<0.001
BMI(kg/m^2^ )	24.84(22.55-27.46)	22.43(20.81-23.70)	26.84(25.81-28.40)	23.86(23.05-24.48)	28.65(26.96-30.88)	2296.922	<0.001
Waist circumference (cm)	89(81-96)	80.00(74.00-87.00)	94.00(89.00-99.00)	88.00(84.00-92.00)	99.00(93.00-104.00)	1641.133	<0.001
SBP (mmHg)	124.0(114.0-136.0)	120.00(109.00-129.00)	127.00(117.00-137.00)	128.50(116.00-137.00)	132.00(121.00-143.50)	331.834	<0.001
DBP (mmHg)	77.0(69.3-87.0)	73.00(66.00-81.00)	80.00(72.00-88.00)	78.50(72.75-88.00)	84.00(75.00-92.00)	325.173	<0.001
FBG (mmol/L)	5.37(5.14-5.73)	5.32(5.10-5.60)	5.38(5.18-5.74)	5.49(5.16-5.90)	5.47(5.18-5.92)	79.428	<0.001
TC (mmol/L)	4.57(3.92-5.19)	4.44(3.83-5.03)	4.56(3.99-5.20)	4.79(4.00-5.42)	4.83(4.16-5.45)	64.978	<0.001
HDL-C(mmol/L)	1.28(1.08-1.54)	1.45(1.22-1.73)	1.23(1.05-1.44)	1.19(1.03-1.45)	1.10(0.94-1.25)	561.377	<0.001
LDL-C(mmol/L)	3.02(2.50-3.58)	2.86(2.40-3.45)	3.05(2.56-3.61)	3.185(2.68-3.76)	3.25(2.70-3.80)	93.608	<0.001
TG (mmol/L)	1.42(0.97-2.15)	1.10(0.81-1.58)	1.50(1.10-2.17)	1.83(1.37-2.51)	2.12(1.51-3.01)	614.575	<0.001
ALT (U/L)	23.0(16.0-33.0)	18.0(13.0-15.0)	23.0(17.0-32.0)	30.50(22.0-41.75)	35.0(25.0-53.0)	705.754	<0.001
BUA (µmol/L)	334.0(276.0-394.0)	298.0(248.0-354.0)	342.0(291.0-397.0)	363.5(313.25-419.0)	387.09(335.5-444.0)	519.812	<0.001
TyG index	5.59(5.20-6.05)	5.33(5.01-5.690)	5.67(5.31-6.08)	5.90(5.55-6.26)	6.04(5.66-6.42)	649.744	<0.001

BMI, body mass index; SBP, systolic blood pressure; DBP, diastolic blood pressure; FBG, fasting blood glucose; TGs, triglycerides; TC, total cholesterol; HDL-c, high-density lipoprotein cholesterol; LDL-c, low-density lipoprotein cholesterol; ALT, alanine aminotransferase; BUA, blood uric acid; TyG index, a product of triglycerides and fasting glucose. The continuous data were expressed as median and interquartile range (IQR). Kruskal-Wallis H test or Mann-Whitney U test was used for comparison between groups. The categorical variables are expressed as percentages. Chi-square test was used to compare categorical variables.

### 3.2 Incidence of type 2 diabetes in subjects with or without NAFLD

The follow-up period was 104 weeks (2.0 years). The results are shown in **Table 2**. The incidence rate of T2DM was 1.72% (24/1398) in the nonoverweight without NAFLD group, 11.88% (19/160) in the nonoverweight with NAFLD group, 5.01% (38/758) in the overweight without NAFLD group and 12.70% (87/658) in the overweight with NAFLD group. The number of participants with incident T2DM was larger in the NAFLD group than in the non-NAFLD group. The risk rate of type 2 diabetes was higher in the nonalcoholic fatty liver group than in the non-NAFLD group. In the case of unadjusted age, sex and other risk factors, the subjects in the lean with NAFLD group and overweight or obese with NAFLD group had HRs of 7.23 (95% confidence interval (CI) 3.96–13.20) and 7.77 (95% confidence interval (CI) 4.95–12.21), respectively, for the development of diabetes compared with those in the lean without NAFLD group. After adjusting for the above risk factors, the subjects in the lean with NAFLD group and overweight or obesity with NAFLD group had HRs of 3.83 (95% confidence interval (CI) 2.02-7.24) and 3.84 (95% confidence interval (CI) 2.28-6.47), respectively, for the development of diabetes compared with those in the lean without NAFLD group (p<0.001). The results suggested that the risk of developing type 2 diabetes was similar in the two groups.

**Table 2:** Subgroup analysis of the Cox proportional hazard model for the incidence of type 2 diabetes from the 3001 subjects with NAFLD and the subjects without NAFLD.

Grouping of subjects	No of subjects	No of subjects Who developed diabetes (%)	Hazard Ratio (95% CI)	
			Model 1	Model 2
Lean without NAFLD	1398	24(1.72)	1	1
Over weight (or obesity) without NAFLD	758	38(5.01)	2.97(1.78-4.95) **	1.80 (1.05–3.08) *
Lean with NAFLD	160	19(11.88)	7.23(3.96–13.20) **	3.83(2.02-7.24) **
Over weight (or obesity) with NAFLD	685	87(12.70)	7.77(4.95–12.21) **	3.84(2.28-6.47) **

Model 1 Risk factors were unadjusted; Model 2 adjusted for age, sex, TC, LDL-C, HDL-C, SBP, DBP, ALT, BUA, and TyG index.

NAFLD, nonalcoholic fatty liver disease; BMI, body mass index; TC, total cholesterol; LDL-C. low density lipoprotein cholesterol: HDL-C. High-density lipoprotein cholesterol; SBP, systolic blood pressure; DBP, diastolic blood pressure; ALT, alanine aminotransferase; BUA, blood uric acid; TyG index, a product of triglyceride and fasting glucose; *p<0.05; **p<0.001, by chi-square test.

### 3.3 The cumulative hazard ratios of incident T2DM and the results of Kaplan−Meier survival analysis

**Figure 2:**
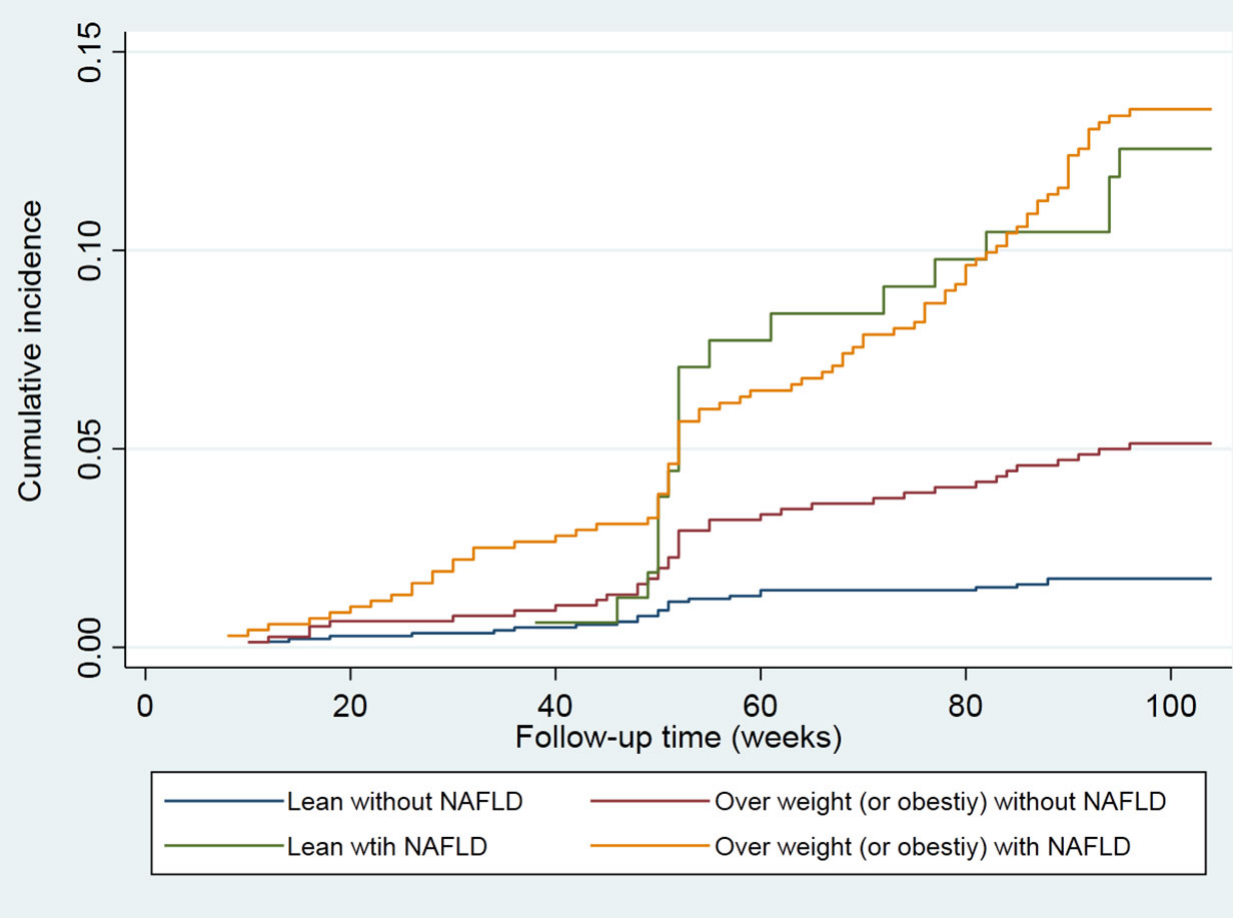
The cumulative hazard ratios of incident T2DM in different groups.

The cumulative hazard ratios of incident T2DM are indicated in **Figure 2**. Univariate COX regression results according to the presence or absence of nonalcoholic fatty liver disease and overweight or obesity groups showed that both NAFLD and overweight/obesity were significantly associated with an increased risk of incident T2DM. Lean subjects without NAFLD were taken as the reference group, and the Logran method was used to compare the differences in the distribution of “survival” (pairwise comparisons of differences in the incidence of type 2 diabetes) among the four groups. The incidence of type 2 diabetes was the same in the lean with NAFLD group and the overweight/obesity with NAFLD group (3.83 vs 3.84, p=0.778). The incidence of type 2 diabetes was significantly different among the other groups, p<0.01 (**Table 3**).

**Table 3:** Log rank (Mantel−Cox) test results of paired comparisons.

Grouping of subjects	N1		N2		N3		N4	
	*X* ^2^ *-value*	*P-value*	*X^2^-value*	*P-value*	*X^2^-value*	*P-value*	*X^2^-value*	*P -value*
N1			19.097	.000	56.323	.000	111.138	.000
N2	19.097	.000			10.699	.001	26.583	.000
N3	56.323	.000	10.699	.001			.079	.778
N4	111.138	.000	26.583	.000	.079	.778		

N1, lean without NAFLD; N2, overweight (or obesity) without NAFLD; N3, lean with NAFLD; N4, overweight (or obesity) with NAFL.

### 3.4 Results of COX regression subgroup analysis of lean nonalcoholic fatty liver disease and risk of T2DM

According to the level of waist circumference, the study population was divided into a normal waist circumference group (man<90 cm, woman<80 cm) and an excessive waist circumference group (man≥90 cm, woman≥80 cm). In the normal waist circumference group, the lean without NAFLD group was used as the reference group. After adjusting for risk factors such as age, sex and blood pressure, the overweight or obesity without NAFLD group and lean with NAFLD group had a risk of type 2 diabetes of 0.60 (0.14-2.66) and 1.93 (0.70-5.35), respectively (p>0.05), and the overweight or obesity with NAFLD group had a risk of type 2 diabetes of 4.20 (1.44-12.22), p<0.01, while in the excessive waist circumference group. After adjusting for risk factors such as age, sex and blood pressure, the lean without NAFLD group was taken as the reference group. The lean with NAFLD group and overweight (or obesity) with NAFLD group had a risk of type 2 diabetes of 3.88 (1.56-9.66) and 3.3 (1.52-7.14), respectively (p<0.01), while the overweight or obesity without NAFLD group had a risk of type 2 diabetes of 1.80 (0.82-3.93), p>0.05 (**Table 4**).

**Table 4:** Rates of incident and hazard ratio of type 2 diabetes based on waist circumference of the 3001 subjects with NAFLD and the subjects without NAFLD.

By waist circumference,	No of subjects	No of subjects who developed diabetes (%)	Hazard Ratio (95% CI)	
			Model 1	Model 2
Normal waist circumference (man<90 cm, woman<80 cm)				
Lean without NAFLD	1099	16(1.46)	1	1
Over weight (or obesity) without NAFLD	139	2(1.44)	0.99(0.23-4.30) #	0.60 (0.14-2.66) #
Lean with NAFLD	78	7(8.97)	6.35(2.61-15.44) **	1.93(0.70-5.35) #
Over weight (or obesity) with NAFLD	44	5(11.36)	8.08(2.96-22.07) **	4.20(1.44-12.22) *
Excess waist circumference (man≥90 cm, woman≥80 cm)				
Lean without NAFLD	298	8(2.68)	1	1
Over weight (or obesity) without NAFLD	619	36(5.82)	2.21(1.03-4.74) *	1.80(0.82-3.93) #
Lean with NAFLD	82	12(14.63)	5.70(2.33-13.95) **	3.88(1.56-9.66) *
Over weight (or obesity) with NAFLD	641	82(12.79)	5.0(2.42-12.34) **	3.30(1.52-7.14) *

Model 1 Risk factors were not adjusted; Model 2 adjusted for age, sex, TC, LDL-C, HDL-C, SBP, DBP, ALT, BUA, and TyG index.

NAFLD, nonalcoholic fatty liver disease; BMI, body mass index; TC, total cholesterol; LDL-C. Low-density lipoprotein cholesterol: HDL-C, high-density lipoprotein cholesterol; SBP, systolic blood pressure DBP, diastolic blood pressure; ALT, alanine aminotransferase; BUA, blood uric acid; TyG index, a product of triglyceride and glucose index; #p>0.05; *p<0.01; **p<0.001, by chi-square test.

## 4 Discussion

In our present study, among 3001 eligible participants, 28.16% had NAFLD, and 5.33% had lean NAFLD. In the NAFLD population, 18.93% had lean NAFLD. The detection rate of NAFLD was 10.27% in those with BMI<25 kg/m^2^ and 45.6% in those with BMI ≥ 25 kg/m^2^. Our results are similar to those of previous studies. In a recent study from the United States, Zou B et al. found that the overall prevalence of NAFLD was 32.3%. Among patients with NAFLD, 29.7% were nonobese, and 13.6% had lean NAFLD (23). A large meta-analysis covering 84 studies worldwide showed that 19.2% of the subjects in the NAFLD population were lean, 40.8% were nonobese, and the prevalence of nonobese NAFLD and lean NAFLD was 12.1% and 5.1%, respectively (14). ShiY et al. reported a meta-analysis of 55,936 lean/nonobese subjects, and the total prevalence of NAFLD in lean and nonobese subjects was 10.2% and 15.7%, respectively (24). Zou ZY et al. reported an overall prevalence of NAFLD of 14.5% in a meta-analysis that included 155,846 nonobese participants (25). The prevalence of lean NAFLD increased between 1988 and 2017. Results of a meta-analysis of 33 observational studies involving 205,307 individuals from 14 countries. The global prevalence of lean NAFLD was 4.1% (95% CI: 3.4-4.8%). Among lean subjects, the prevalence of NAFLD was 9.7% (95% CI: 7.7-11.8%), and Asians had the highest prevalence of lean NAFLD (4.8%, 95% CI: 4.0-5.6%) (26). **Table 1** shows that both overweight and obese NAFLD and lean NAFLD have much higher metabolic characteristics than those without NAFLD, and the risk of metabolic diseases is correspondingly increased

Among the 4 groups in the present study, the lean with NAFLD group had the oldest average age and male predominance (85.0%). BMI and waist circumference were higher than those in the lean without NAFLD group, and fasting blood glucose was the highest. Systolic blood pressure, serum total cholesterol, low-density lipoprotein, triglyceride and blood uric acid levels were also higher than those in the non-NAFLD group, and high-density lipoprotein was lower than those in the non-NAFLD group. The results of this study showed that the metabolic index value level of the lean with NAFLD group was basically the same as that of the overweight (or obesity) with NAFLD group, which was the same as the results of other similar studies (13, 27).

Insulin resistance (IR) is not only the main pathogenesis of obese nonalcoholic fatty liver disease but also plays a key role in the pathogenesis of lean NAFLD (28). Studies have shown that the triglycerides and glucose index (TyG index) could be a reliable surrogate index for IR (18, 29), and the results of this study suggested that the insulin resistance level of people with lean NAFLD was higher than that of lean without NAFLD and overweight (or obesity) without NAFLD and was slightly lower than that of those with overweight (or obesity) with NAFLD. Our study suggests that insulin resistance plays an important role in the pathogenesis of lean fatty liver disease and type 2 diabetes mellitus.

To date, there are few studies on the association between lean fatty liver and the risk of type 2 diabetes, and the definition of lean fatty liver is based on BMI<25.0 kg/m^2^ or <23.0 kg/m^2^ without waist circumference stratification. Fukuda T and his colleagues had shown that ‘A cutoff point of BMI 23 kg/m^2^ was used to define overweight (≥23.0 kg/m^2^) or nonoverweight (<23.0 kg/m^2^). This was a population-based retrospective cohort study of 4629 participants who were enrolled in a health check-up program for a mean follow-up of 12.8 years. The adjusted hazard ratios for incident T2DM compared with the nonoverweight without NAFLD group were as follows: 3.59 (95% CI: 2.14–5.76) in the nonoverweight with NAFLD group, 1.99 (95% CI: 1.47–2.69) in the overweight without NAFLD group and 6.77 (95% CI: 5.17–8.91) in the overweight with NAFLD group. The adjusted hazard ratio in the nonoverweight with NAFLD group was significantly higher than that in the overweight without NAFLD group or that in the nonoverweight without NAFLD group (30). Another cohort study from the Japanese Physical Examination Population Database (JPEPD) with an average follow-up of 6 years showed that after adjusting for confounding factors, the fully adjusted HR (95% CI) for incident diabetes in lean NAFLD vs lean without NAFLD patients was 2.58 (95% CI: 1.68 -3.97) in the study population as a whole or in subgroups stratified by sex, and the risk of type 2 diabetes was the same for lean and overweight or obese with NAFLD (31).

In our study, lean nonalcoholic fatty liver was defined as a BMI of less than 25.0 kg/m^2^. If waist circumference was not stratified, after adjusting for related risk factors, compared with nonalcoholic fatty liver with BMI<25.0 kg/m^2^. The adjusted hazard ratios for incident T2DM were 3.83 (2.02-7.24) in the lean with NAFLD group, 1.80 (1.05–3.08) in the overweight or obesity without NAFLD group and 3.84 (2.28-6.47) in the overweight or obesity with NAFLD group. Lean nonalcoholic fatty liver group than in the study of the risk of type 2 diabetes in the Japanese population is higher, the reason is that we study the lean nonalcoholic fatty liver disease in cutting point than their high, but lean nonalcoholic fatty liver disease group and overweight fuelling nonalcoholic fatty liver disease is the same risk for type 2 diabetes (3.83 vs3.84). After stratification by waist circumference, this study found that in the normal waist circumference group, compared with the BMI<25.0 kg/m^2^ and nonalcoholic fatty liver groups, the hazard ratio of type 2 diabetes in the lean nonalcoholic fatty liver group was 1.93 (0.70-5.35, P>0.05) after adjusting for related risk factors. In the overweight waist circumference group (abdominal obesity group), the risk ratio of type 2 diabetes in the lean nonalcoholic fatty liver group was 3.88 (1.56-9.66), P<0.05. The results of this study showed that overweight nonalcoholic fatty liver disease is an independent risk factor for type 2 diabetes in the presence of a normal waist circumference, while lean nonalcoholic fatty liver disease is not an independent risk factor for type 2 diabetes. The high incidence of type 2 diabetes in people with lean nonalcoholic fatty liver disease may be due to the higher level of insulin resistance, higher blood lipid levels, and different degrees of steatohepatitis in this group.

In the presence of excess waist circumference (abdominal obesity), lean nonalcoholic fatty liver disease was an independent risk factor for type 2 diabetes, and the risk of type 2 diabetes was slightly higher than that of overweight and obese nonalcoholic fatty liver disease (3.88 vs 3.30). In abdominal obesity, there is an increase in visceral fat, which is a major source of free fatty acids and inflammatory cytokines. Increased levels of visceral fat can lead to insulin resistance and type 2 diabetes. Feng RN et al. found that abdominal obesity was closely related to type 2 diabetes in Chinese adults (32). NAFLD is strongly associated with the pathogenesis of type 2 diabetes mellitus. NAFLD is a multisystem disease characterized by “ectopic fat accumulation” in the liver, leading to a series of pathophysiological manifestations. When hepatic fat accumulation occurs, long-chain fatty acids (LCFAs) and triglycerides 3-phosphate (derived from glycolysis) form monoacylglycerol, diacylglycerol (DAG), and triacylglycerol (TAG) within hepatocytes. Lipid synthesis can increase the production of intermediates, such as diacylglycerol DAG, dipalmitoyl phosphate (Di-P PA), and other lipid products, such as ceramides. Increased production of these lipid products, especially DAG, leads to “resistance” within the hepatic insulin signaling pathway, and ceramide inhibits distal insulin signaling, which is also a mediator of inflammation and oxidative stress. In addition, liver fat accumulation can secrete hepatokines, which affect the insulin signaling pathway and subclinical inflammation, cause hepatic/peripheral insulin resistance and promote liver inflammation (33, 34). NAFLD often coexists with metabolic syndrome (MS) or predisposes patients to metabolic diseases. Therefore, the 2020 International Panel of Experts recommended that NAFLD be renamed metabolically associated fatty liver disease (MAFLD). The diagnostic criteria of MAFLD are based on histological (liver biopsy), abdominal imaging, and blood biomarker evidence of hepatic fat accumulation (hepatocellular steatosis). Combined with one of the following three conditions: overweight/obesity, type 2 diabetes mellitus and at least two risk factors for metabolic abnormalities (35), MAFLD is prone to develop into type 2 diabetes mellitus. According to the diagnostic criteria of MAFLD, overweight/obese fatty liver can be diagnosed as MAFLD, and lean fatty liver combined with risk factors for metabolic abnormalities also belongs to MAFLD. Ye JZ et al. conducted cohort cluster analysis on the MAFLD population and found that the high abdominal circumference cluster has a higher risk of T2DM and CVD after long-term follow-up, and its pathogenesis is related to the high waist circumference population often accompanied by hyperfree lipasemia and hyperinsulinemia (36).

This study also confirmed that the risk of type 2 diabetes in NAFLD patients with abdominal obesity was significantly higher than that in NAFLD patients with normal waist circumference, regardless of BMI. According to previous studies and the results of this study, regardless of whether it is MAFLD or not, the risk of type 2 diabetes in lean nonalcoholic fatty liver disease is similar. If NAFLD is MAFLD, whether it is lean nonalcoholic fatty liver disease or nonlean nonalcoholic fatty liver disease, it has a high risk of type 2 diabetes. BMI-driven approaches for NAFLD should be replaced by better diagnostic tools emphasizing the assessment of metabolic disorders and advanced liver fibrosis (16). It should be explored which clinical manifestations and outcomes of lean NAFLD meet the criteria for MAFLD and which do not.

There are several limitations of our study. As this study subjects were from healthy people undergoing physical examination, most of them were young and middle-aged people under 60 years old, so the correlation between lean elderly nonalcoholic fatty liver disease and the risk of type 2 diabetes was not covered in this study. Second. Due to the small number of women in the study population, this study was not conducted according to gender classification. Third. It is a single-center retrospective cohort study with a short follow-up period for the study population. In the future, a prospective multicenter cohort study with a longer follow-up period and a larger sample size is needed to strengthen the verification of the results. Fourth. For the reason of health examination, the glucose tolerance test was not used to exclude patients with type 2 diabetes during the baseline survey in this study. Therefore, a very small number of patients with impaired glucose tolerance with fasting blood glucose <6.1 mmol/L may participate in the follow-up study, but we believe that this has no significant impact on the study results. Fifth. Considering the short follow-up time of the present study, smoking, a small amount of alcohol consumption and exercise have little influence on the risk of type 2 diabetes, so the lifestyle data of smoking, alcohol consumption and exercise were not collected when the baseline data were collected.

## 5 Conclusion

The results of this study showed that abdominal obesity was a stronger risk factor for type 2 diabetes than overweight/obesity, with BMI ≥ 25 kg/m^2^ as the cutoff point in nonalcoholic fatty liver disease. In waist circumference normal young and middle-aged people, lean nonalcoholic fatty liver disease (BMI < 25.0 kg/m^2^) is not an independent risk factor for type 2 diabetes. In the abdominal obesity population, lean nonalcoholic fatty liver disease is an independent risk factor for type 2 diabetes and causes the risk of type 2 diabetes and overweight fueling nonalcoholic fatty liver disease to be the same. The risk of lean nonalcoholic fatty liver disease in type 2 diabetes mellitus is affected by the number of risk factors for metabolic abnormalities; among them, the most important factor is abdominal obesity. It is of great significance to classify nonalcoholic fatty liver disease into metabolic fatty liver disease and classify its management and treatment according to the risk factors for type 2 diabetes mellitus, cardiovascular and cerebrovascular diseases and malignancy, as well as cluster risk factors for the prevention and treatment of nonalcoholic fatty liver disease complications.

## Data availability statement

The original contributions presented in the study are included in the article/supplementary material. Further inquiries can be directed to the corresponding authors.

## Ethics statement

The studies involving human participants were reviewed and approved by Medical Research Ethics Committee of Karamay Hospital of Integrated Chinese and Western Medicine. The patients/participants provided their written informed consent to participate in this study.

## Author contributions

NL designed the study and wrote the manuscript. HT contributed to data interpretation and reviewed the manuscript. NL and HT and WX contributed to data Statistical analysis. WX, SW, DL, MC, CX, MZ were involved in data collection and data cleaning. All authors contributed to the article and approved the submitted version.

## References

[1] Targher G Corey KE Byrne CD Roden M. The complex link between NAFLD and type 2 diabetes mellitus - mechanisms and treatments. Nat Rev Gastroenterol Hepatol (2021) 18:599–612. doi: 10.1038/s41575-021-00448-y 33972770

[2] Kuchay MS Martínez-Montoro JI Choudhary NS Fernández-García JC Ramos- Molina. Non-alcoholic fatty liver disease in lean and non-obese individuals: current and future challenges. Biomedicines (2021) 9(10):1346. doi: 10.3390/biomedicines9101346 34680463 PMC8533092

[3] Younossi ZM Koenig AB Abdelatif D Fazel Y Henry L Wymer M.. Global epidemiology of nonalcoholic fatty liver disease–meta analytic assessment of prevalence, incidence, and outcomes. Hepatology (2016) 64:73–84. doi: 10.1002/hep.28431 26707365

[4] Huang DQ El-Serag HB Loomba R. Global epidemiology of NAFLD-related HCC: trends, predictions, risk factors and prevention. Nat Rev Gastroenterol Hepatol (2021) 18:223–38. doi: 10.1038/s41575-020-00381-6 PMC8016738 33349658

[5] European Association for the Study of the Liver (EASL). European Association for the study of diabetes (EASD); European association for the study of obesity (EASO). EASL-EASD EASO clinical practice guidelines for the management of nonalcoholic fatty liver disease. Diabetologia (2016) 59(6):1121–40. doi: 10.1007/s00125-016-3902-y 27053230

[6] Smith BW Adams LA. Nonalcoholic fatty liver disease. Crit Rev Clin Lab Sci (2011) 48:97–113. doi: 10.3109/10408363.2011.596521 21875310

[7] Targher G Tilg H Byrne CD. Non-alcoholic fatty liver disease: a multisystem disease requiring a multidisciplinary and holistic approach. Lancet Gastroenterol Hepatol (2021) 6(7):578–88. doi: 10.1016/S2468-1253(21)00020-0 33961787

[8] Byrne CD Targher G. NAFLD: a multisystem disease. J Hepatol (2015) 62:S47–64. doi: 10.1016/j.jhep.2014.12.012 25920090

[9] Younossi ZM Golabi P Avila L Paik JM Srishord M Fukui N. The global epidemiology of NAFLD and NASH in patients with type 2 diabetes: a systematic review and meta-analysis. J Hepatol (2019) 71(4):793–801. doi: 10.1016/j.jhep.2019.06.021 31279902

[10] Younossi ZM Stepanova M Afendy M Fang Y Younossi Y Mir H. Changes in the prevalence of the most common causes of chronic liver diseases in the united states from 1988 to 2008. Clin Gastroenterol Hepatol (2011) 9:524–30. doi: 10.1016/j.cgh.2011.03.020 21440669

[11] Qiu S Cai X Sun Z Li L Zügel M Steinacker JM. Association between physical activity and risk of nonalcoholic fatty liver disease: a meta-analysis. Therap Adv Gastroenterol (2017) 10:701–13. doi: 10.1177/1756283X17725977 PMC5598813 28932271

[12] Lonardo A Lugari S Ballestri S Nascimbeni F Baldelli E Maurantonio M.. A round trip from nonalcoholic fatty liver disease to diabetes: molecular targets to the rescue? Acta Diabetologica (2019) 56(4):385–96. doi: 10.1007/s00592-018-1266-0 30519965

[13] Wang A Dhaliwal J Mouzaki M. Lean nonalcoholic fatty liver disease. Clinica Nutr (2019) 38(3):975–81. doi: 10.1016/j.clnu.2018.08.008 30466956

[14] Ye Q Zou B Yeo YH Li J Huang DQ Wu Y. Global prevalence, incidence, and outcomes of nonobese or lean nonalcoholic fatty liver disease: a systematic review and meta-analysis. Lancet Gastroenterol Hepatol (2020) 5(8):739–52. doi: 10.1016/S2468-1253(20)30077-7 32413340

[15] Younes R Govaere O Petta S Miele L Tiniakos D Burt A. Caucasian Lean subjects with nonalcoholic fatty liver disease share long-term prognosis of nonlean: time for reappraisal of BMI-driven approach? Gut (2022) 71(2):382–90. doi: 10.1136/gutjnl-2020-322564 33541866

[16] Ren TY Fan JG. What are the clinical settings and outcomes of lean NAFLD? Nat Rev Gastroenterol Hepatol (2021) 18(5):289–90. doi: 10.1038/s41575-021-00433-5 33649583

[17] Amedeo L Fabio N Alessandro M Giovanni T. Hypertension, diabetes, atherosclerosis and NASH: cause or consequence? J Hepatol (2018) 68(2):335–52. doi: 10.1016/j.jhep.2017.09.021 29122390

[18] Simental-Mendia LE Rodriguez-Moran M Guerrero-Romero F. The product of fasting glucose and triglycerides as surrogate for identifying insulin resistance in apparently healthy subjects. Metab Syndr Relat Disord (2008) 6:299–304. doi: 10.1089/met.2008.0034 19067533

[19] Saadeh S Younossi ZM Remer EM Gramlich T Ong JP Hurley M. The utility of radiological imaging in nonalcoholic fatty liver disease. Gastroenterology (2002) 123(3):745–50. doi: 10.1053/gast.2002.35354 12198701

[20] Chalasani N Younossi Z Lavine JE Charlton M Cusi K Rinella M. The diagnosis and management of nonalcoholic fatty liver disease:Practice guidance from the American association for the study of liver diseases. Hepat (2018) 67 (1):328–57. doi: 10.1002/hep.29367 28714183

[21] WHO. Defnition, diagnosis and classifcation of diabetes mellitus and its complications-Part1:diagnosis and classifcation of diabetes mellitus. (Geneva: WHO) (1999).

[22] Misra A Vikram NK Gupta R Pandey RM Wasir JS Gupta VP.. Waist circumference cut off points and action levels for Asian indians for identification of abdominal obesity. Int J Obes (Lond) (2006) 30:106–11. doi: 10.1038/sj.ijo.0803111 16189502

[23] Zou B Yeo YH Nguyen VH Cheung R Ingelsson E Nguyen MH.. Prevalence, characteristics and mortality outcomes of obese, nonobese and lean NAFLD in the united states, 1999–2016. J Intern Med (2020) 288:139–51. doi: 10.1111/joim.13069 32319718

[24] Shi Y Wang Q Sun Y Zhao X Kong Y Ou X. The prevalence of Lean/Nonobese nonalcoholic fatty liver disease: a systematic review and meta-analysis. J Clin Gastroenterol (2020) 54:378–87. doi: 10.1097/MCG.0000000000001270 31651571

[25] Zou ZY Wong VWS Fan JG. Epidemiology of nonalcoholic fatty liver disease in non-obese populations: meta-analytic assessment of its prevalence, genetic, metabolic, and histological profifiles. J Dig Dis (2020) 21:372–84. doi: 10.1111/1751-2980.12871. 32369237

[26] Lu FB Zheng KI Rios RS Targher G Byrne CD Zheng MH.. Global epidemiology of lean non-alcoholic fatty liver disease: a systematic review and meta-analysis. J Gastroenterol Hepatol (2020) 35:2041–50. doi: 10.1111/jgh.15156 32573017

[27] Niriella MA Kasturiratne A Pathmeswaran A De Silva ST Perera KR Subasinghe SKCE. Lean non-alcoholic fatty liver disease (lean NAFLD): characteristics, metabolic outcomes and risk factors from a 7-year prospective, community cohort study from Sri Lanka. Hepatol Int (2019) 13(3):314–22. doi: 10.1007/s12072-018-9916-4 30539516

[28] Bugianesi E Gastaldelli A Vanni E Gambino R Cassader M Baldi S. Insulin resistance in nondiabetic patients with nonalcoholic fatty liver disease: sites and mechanisms. Diabetologia (2005) 48:634–42. doi: 10.1007/s00125-005-1682-x 15747110

[29] Guerrero-Romero F Simental-Mendia LE Gonzalez-Ortiz M Martínez-Abundis E Ramos-Zavala MG Hernández-González SO. The product of triglycerides and glucose, a simple measure of insulin sensitivity. comparison with the euglycemic-hyperinsulinemic clamp. J Clin Endocrinol Metab (2010) 95:3347–51. doi: 10.1210/jc.2010-0288 20484475

[30] Fukuda T Hamaguchi M Kojima T Hashimoto Y Ohbora A Kato T. The impact of nonalcoholic fatty liver disease on incident type 2 diabetes mellitus in nonoverweight individuals. Liver Int (2016) 36:275–83. doi: 10.1111/liv.12912 26176710

[31] Wei L Cheng X Luo Y Yang R Lei Z Jiang H. Lean nonalcoholic fatty liver disease and risk of incident diabetes in a euglycemic population undergoing health check-ups: a cohort study. Diabetes Metab (2021) 47(3):101200. doi: 10.1016/j.diabet.2020.08.008 33075504

[32] Feng RN Zhao C Wang C Niu YC Li K Guo FC. BMI is strongly associated with hypertension, and waist circumference is strongly associated with type 2 diabetes and dyslipidemia, in northern Chinese adults. J Epidemiol. (2012) 22:317–23. doi: 10.2188/jea.JE20110120 PMC3798650 22672914

[33] Byrne CD. Ectopic fat, insulin resistance and nonalcoholic fatty liver disease. Proc Nutr Soc (2013) 72:412–9. doi: 10.1017/S0029665113001249 23668723

[34] Byrne CD. Dorothy Hodgkin Lecture 2012: nonalcoholic fatty liver disease,insulin resistance and ectopic fat: a new problem in diabetes management. Diabetes Med (2012) 29:1098–107. doi: 10.1111/j.1464-5491.2012.03732.x 22672330

[35] Eslam M Newsome PN Sarin SK Anstee QM Targher G Romero-Gomez M. A new defifinition for metabolic dysfunction-associated fatty liver disease: an international expert consensus statement. J Hepatol (2020) 73(1):202–9. doi: 10.1016/j.jhep.2020.03.039 32278004

[36] Ye JZ Zhuang XD Li X Gong X Sun Y Wang W. Novel metabolic classification for extrahepatic complication of metabolic associated fatty liver disease: a data-driven cluster analysis with international validation. Metabolism (2022) 136:155294. doi: 10.1016/j.metabol.2022.155294 35995280

